# Component Adjustment of Poly(arylene ether nitrile) with Sulfonic and Carboxylic Groups for Dielectric Films

**DOI:** 10.3390/polym11071135

**Published:** 2019-07-03

**Authors:** Chenchen Liu, Shuning Liu, Jian Lin, Lingling Wang, Yumin Huang, Xiaobo Liu

**Affiliations:** Research Branch of Advanced Functional Materials, School of Materials and Energy, University of Electronic Science and Technology of China, Chengdu 611731, China

**Keywords:** poly(arylene ether nitrile), sulfonic group, carboxylic group, dielectric properties

## Abstract

Poly(arylene ether nitrile)s with sulfonic and carboxylic groups (SCPEN) were synthesized to investigate their electrical properties. This new series of copolymers were prepared by copolymerization of phenolphthalein, potassium hydroquinonesulfonate, and 2,6-difluorobenzonitrile, in different mole ratios. Their thermal, mechanical and dielectric properties were investigated in detail. By adjusting the composition of sulfonic and carboxylic groups, it can be concluded that the dielectric constant increases with the increase of sulfonic groups, and mechanical and thermal properties improve with the increase of carboxylic groups. The as-prepared SCPEN films show potential applications in electronic storage materials, which provide insights into the correlation of SCPEN electrical properties with its chemical structure. The structure–property relationship is established to broaden the application of functionalized PEN. Furthermore, SCPEN with rich polar groups may also be used as the polymer matrix to increase the interaction with the filler surface, ensuring a better dispersion of filler in the matrix. This provides a reference for the development of high dielectric materials.

## 1. Introduction

With the rapid development of modern society, there is an increasing demand for breakthroughs in new dielectric materials in diverse applications including capacitors, actuators, gate dielectrics, and energy-storage devices [1]. Among all dielectric materials, polymer dielectric materials have attracted wide attention owing to their lightweight, flexibility and ease of processing [2]. However, the intrinsic low dielectric constant of polymers often limits their use. It is a common strategy to prepare polymer composites with good dielectric properties by adding fillers such as ceramic fillers [3,4], conductive fillers [5,6] and semi-conductive fillers [7,8]. However, the resulting polymer composites tend to deteriorate in mechanical and processing properties as the addition of rigid particles. Moreover, poor compatibility in the interfacial region of organic-inorganic composites results in high dielectric loss and a low dielectric breakdown field [9]. Molecular design of the polymer is another strategy to increase its intrinsic dielectric constant and keep low dielectric loss. Concretely, the increase of the dielectric constant can be achieved by increasing various polarizations, namely, electron polarization, atomic polarization (vibration polarization), orientation polarization (dipole polarization) and space charge polarization [10]. On the other hand, the dielectric constant of the polymer matrix also has a great influence on the dielectric constant of the polymer composites. The dielectric constant of polymer composites can be improved by increasing the dielectric constant of polymers. Therefore, it is particularly important to design the polymer with a high dielectric constant.

Poly(arylene ether nitrile) (PEN), as a new type of high-performance thermoplastic, exhibits thermal stability, chemical resistance, as well as mechanical properties [11,12,13]. Although there are plenty of polar –CN groups in its side chains, the dielectric constant of most PEN is about 3–4. In order to further increase the dielectric constant, the introduction of more different polar groups into the PEN is expected to effectively increase the dipole polarization. Sulfonic acid and carboxylic acid are common polar groups. In the past years, the polymers containing polar sulfonic groups (–SO_3_H)/carboxylic groups (–COOH) have attracted much attention. For example, the polymers containing sulfonic groups have been widely studied as proton exchange membranes [14,15,16]. Also, sulfonated polymers have also been used to prepare polymer-based dielectric composite materials owing to its high dielectric constant [17,18]. However, high sulfonic acid content may lead to high dielectric loss and poor mechanical properties. It is very necessary to introduce other polar groups to adjust the properties of polymers. Polymers containing carboxylic acid have shown interesting properties, such as adsorption separation, rare earth metal coordination [19,20], etc. Because of the smaller size of the carboxylate anion and weaker acidity of the carboxylic groups, there are strong hydrogen bonds between polymer chains and accordingly excellent physical stability [21]. Moreover, carboxylic groups have reactivity to form grafted or cross-linked polymers [22,23]. It was expected that the properties of the polymer could be well balanced by introducing sulfonic groups and carboxylic groups into the polymer at the same time. Furthermore, the polymers with –SO_3_H or –COOH groups can also be used to modify fillers to improve the dispersion of fillers in the polymer matrix [24,25]. However, to our best knowledge, the electrical properties of PEN bearing both -SO_3_H and –COOH have not been previously studied. Therefore, understanding these electrical properties can diversify the applicability of this material in emerging technologies.

In this study, a series of PEN with different sulfonic and carboxyl groups (SCPEN) are synthetized by polycondensation reaction, and then their electrical properties are evaluated. Besides, the effects of sulfonic groups and carboxyl groups in SCPEN on the thermal properties, mechanical properties and microstructures are also investigated in detail. Furthermore, a correlation between the properties of the polymer and its chemical structure is established, which will broaden the application of functionalized PEN.

## 2. Materials and Methods

### 2.1. Materials

2,6-difluorobenzonitrile (DFBN, AR) and potassium hydroquinonesulfonate (SHQ, AR) were supplied by Sigma Aldrich (Shanghai, China). Phenolphthalein (PP, AR), anhydrous potassium carbonate (K_2_CO_3_, AR), *N*-methyl pyrrolidone (NMP, AR), toluene (AR), ethanol (AR), hydrochloric acid (AR), and *N*,*N*-Dimethylacetamide (DMAc, AR) were supplied by Chengdu Kelong Chemical Co. (Chengdu, China). All the materials above were used as received without further purification. The synthesis of phenolphthalin (PPL) from PP was implemented according to the literature [26].

### 2.2. Preparation of SCPEN Copolymers

The SCPEN copolymers with different ratios of sulfonic groups and carboxylic groups were synthesized according to our previous work [26]. The representative synthesis procedure of SCPEN-50 was as follows: a mixture of SHQ (11.4 g, 0.05 mol), PPL (16 g, 0.05 mol), K_2_CO_3_ (31 g, 0.225 mol) and DFBN (13.9 g, 0.1 mol) were added to a 250 mL three-necked flask equipped with a condenser, a Dean–Stark trap and a mechanical stirrer, followed by adding toluene (25 mL) and NMP (65 mL) as the solvent. The mixture was heated to 140 °C under moderate stirring for 3 h for full dehydration. Afterwards, the azeotropic mixture was gradually heated to 160, 180, and 190 °C by releasing the generated water and toluene and kept for 2 h separately. Then the raw product was poured into the ethanol, and the precipitate was produced. The precipitate was smashed and purified three times with hot ethanol and water to remove the unreacted reagents and remnant solvent. After filtration, the residue was acidified by 2N hydrochloric acid. Finally, the product in acid form was obtained by washing with deionized water several times and drying in a vacuum oven at 80 °C for 24 h. Table 1 shows the molar ratio of reactants, and the final products were denoted as SCPEN-90, SCPEN-70, SCPEN-50, and SCPEN-30.

### 2.3. Preparation of SCPEN Films

The SCPEN copolymer (1 g) was dissolved in 10 mL DMAc under heating conditions. The polymer solution was cast on a clean glass plate and dried in an oven under a controlled program of 80, 100 °C (1 h each) and 120, 140, 160, 180 °C (2 h each) to evaporate the solvent. The films with thicknesses of 20–40 μm were obtained.

### 2.4. Characterization

The chemical structure of SCPENs was characterized with FTIR (8400S, Shimadzu, Tokyo, Japan) and UV-Vis (TU-1810, Persee, Beijing, China). Also, the ^1^H NMR spectra (Bruker AV II-400 MHz spectrometer, Karlsruche, Germany) were measured with tetramethylsilane (TMS) as the internal standard in DMSO-*d*_6_ to further confirm the chemical structure. The thermal properties of the samples were detected with TGA (Q50, TA Instruments, New Castle, DE, USA) at a heating rate of 20 °C·min^−1^ and a nitrogen flow rate of 60 mL·min^−1^. The cross-section morphologies of the films were observed on SEM (6490LV, JSM, Tokyo, Japan) at 20 kV. The dielectric performances of the films were investigated on a precision LCR meter (TH 2819A, Tonghui, Changzhou, China). The electrical breakdown strengths of the films were recorded through a dielectric withstand voltage tester (ZJC-50 KV, Zhonghang Shidai, Beijing, China). The polarization-voltage loops were detected at room temperature by a ferroelectric test system (HVI0203-234, Radiant Technology, Albuquerque, NM, USA). The mechanical properties of the films were performed using a universal testing machine (CMT6104, SANS, Shenzhen, China) at a stretching rate of 5 mm·min^−1^. Prior to various testing, the samples were placed into a vacuum oven for 1 h (or longer) at 100 °C to remove absorbed water as much as possible. The ion exchange capacity (IEC), water uptake (WU) and swelling ratio (SR) measurements of SCPEN films were measured according to the literature [27]. The detailed process is in the Supplementary Information.

## 3. Results and Discussion

### 3.1. Synthesis of SCPEN Copolymers

**Scheme 1:**
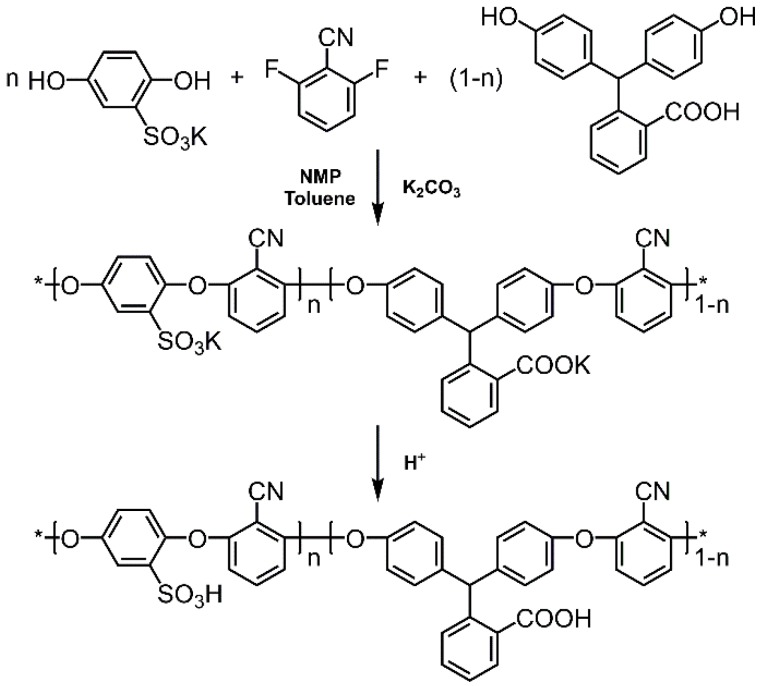
Synthetic route for SCPEN copolymers.

**Figure 1:**
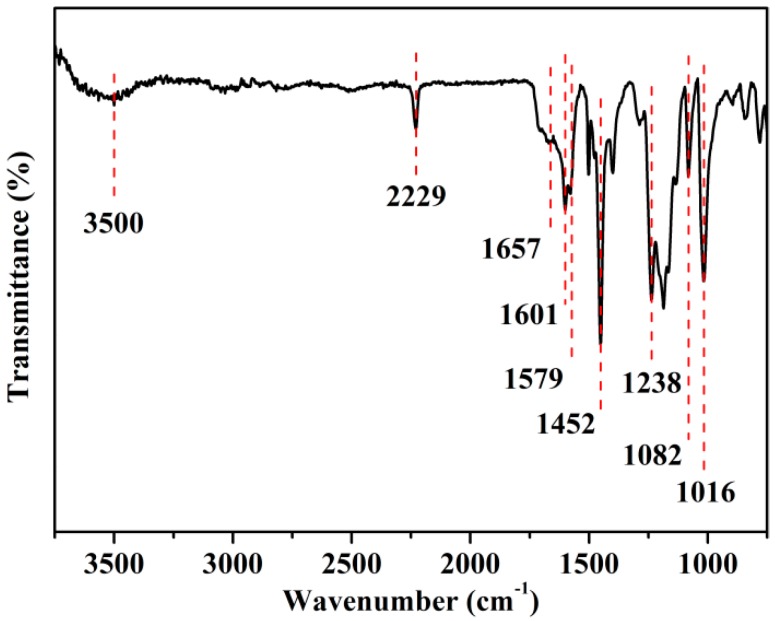
FTIR spectra of SCPEN copolymers.

A series of SCPEN were synthesized by the nucleophilic substitution polycondensation reactions, as displayed in Scheme 1. The components of SCPEN copolymers are shown in Table 1. The chemical structures of SCPENs are identified by FTIR spectra (Figure 1). From the FTIR spectra, it can be informed that the characteristic absorption bands at 1082 and 1016 cm^−^^1^ are referred to as symmetric and asymmetric stretching vibrations of O=S=O in –SO_3_H groups [28]. In addition, the characteristic absorption peaks at 1657 and 1579 cm^−^^1^ are assigned to the bending vibration of –CO– [29]. Moreover, 2229 cm^−^^1^ (–CN), 1601 and 1452 cm^−^^1^ (C=C of Ar) and 1238 cm^−^^1^ (Ar–O–Ar) are assigned to other characteristic absorption peaks of SCPEN [26]. These demonstrate that sulfonic acid groups and carboxylic acid groups have been successfully introduced into poly(arylene ether nitrile).

**Figure 2:**
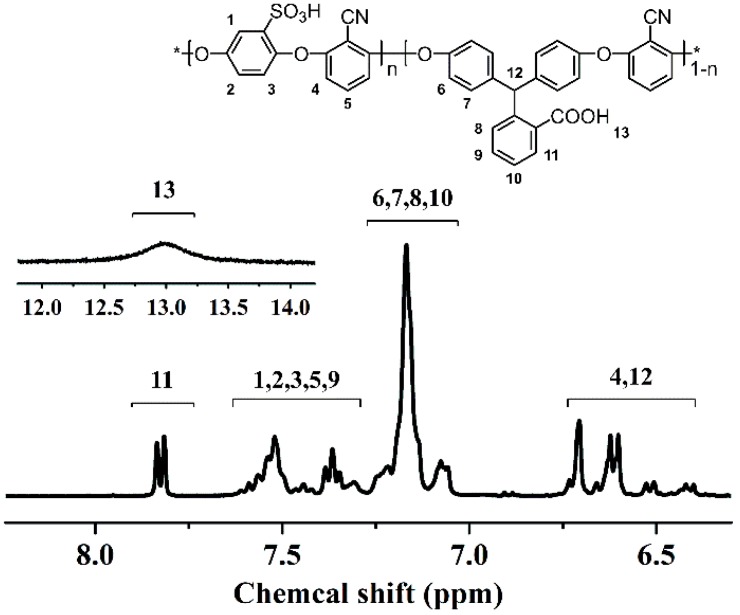
^1^H NMR spectra of SCPEN copolymers.

**Figure 3:**
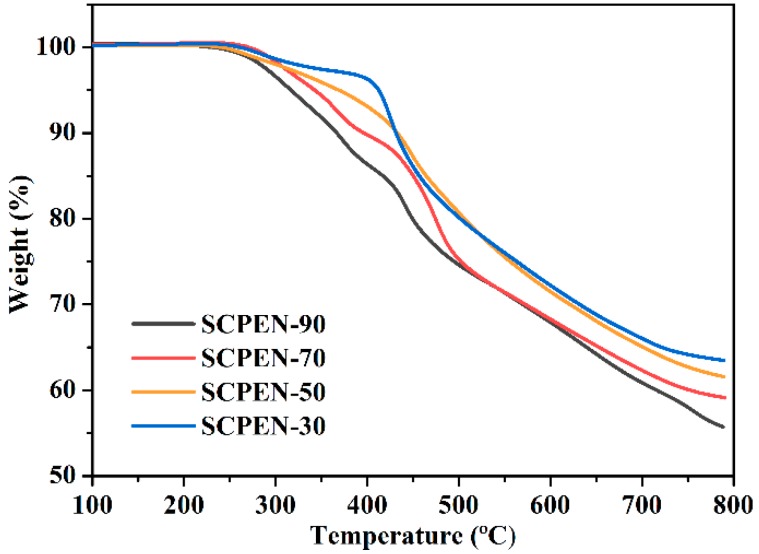
The TGA curves of SCPEN copolymers.

To confirm the structures of SCPENs, the ^1^H NMR spectrum of SCPEN-30 is performed, as shown in Figure 2. It is apparent that the broad peak of carboxyl hydrogen emerged at a high frequency around 13.0 ppm, suggesting the successful introduction of carboxylic groups. As a result of the electron-withdrawing ability of the carboxylic group, a high chemical shift around 7.8 ppm is ascribed to H_11_, which is adjacent to –CO_2_H. The other protons in aromatic rings are also well assigned in Figure 3. Both FTIR and ^1^H NMR spectrum prove that the poly(arylene ether nitrile)s with –SO_3_H and –CO_2_H groups are obtained as desired.

Furthermore, the intrinsic viscosities of the polymers are measured by Ubbelohde viscometer (Liangjing, Shanghai, China). The intrinsic viscosities of the SCPEN copolymers are in the range of 1.10–1.15 (Table 1). This implies that high molecular weight SCPEN copolymers are obtained.

### 3.2. Thermal properties of SCPEN

The thermal properties of the SCPENs under nitrogen atmosphere are evaluated through the TGA experiments, as shown in Table 2 and Figure 3. All the copolymers show two-stage decompositions. The first weight loss at around 250–500 °C is attributed to the decomposition of sulfonic acid and carboxylic acid groups. Another weight loss above 500 °C is due to the decomposition of the copolymer backbones. Moreover, the 5% weight loss temperatures (T_5%_) of all polymers exceed 300 °C, which indicates the excellent thermal stability of SCPEN copolymers. In addition, T_5%_ of SCPENs greatly increases from 317 to 412 °C with the increase of carboxylic acid content (Table 2). It suggests that the polymer contains carboxyl functional groups with better thermal stability than that of the sulfonic acid groups. This could be explained by the introduction of carboxylic acid groups which make the polymer have stronger intermolecular interactions. Likewise, the char yields (CY) at 800 °C are also found to gradually increase with the increasing –COOH contents, from 55.7% for SCPEN-90 to 63.5% for SCPEN-30. All these prove that SPCENs have excellent thermal properties.

### 3.3. Morphology of SCPEN Films

**Figure 4:**
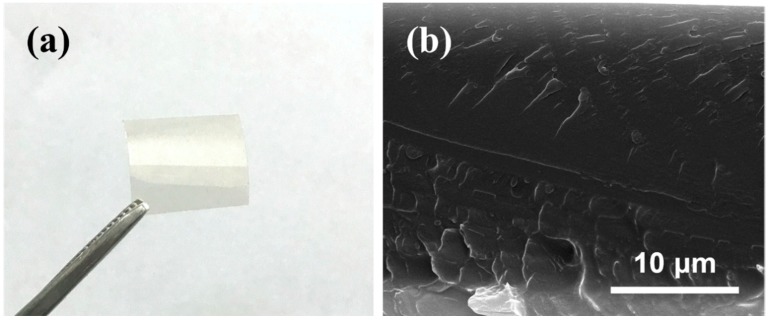
(**a**) The photo and (**b**) cross-sectional SEM images of SCPEN films.

With the SCPEN copolymers in hand, SCPEN films are fabricated by the solution-casting method. From Figure 4a, it can be seen that the yellow transparent SCPEN films are obtained. Also, the films show glossy and flexible. The cross-sectional morphologies of the SCPEN films are visualized by SEM, obtained by brittle fracture in liquid nitrogen. As shown in Figure 4b, it can be intuitively seen that the pure SCPEN film possesses a homogenous section with a smooth and dense surface. It is noteworthy that there are no defects in the cross section of the film, guaranteeing the good performance of the film.

### 3.4. IEC, Water Uptake and Swelling Ratio of SCPEN Films

IEC reflects the density of H^+^ exchangeable functional groups in the film, which is determined by a typical acid-base titration method. The IEC values are related to the content of the sulfonic acid group, as shown in Table 3. In order to check the dimensional stability of the polymer, the water uptake and swelling ratio of the films were studied. With the increase of the carboxylic acid group content, both WU and SR exhibit a downward trend, which is related to the lower IEC values and extensive hydrogen bonds. Meanwhile, high IEC of SCPEN-90 does not encounter excessive swelling degrees in length, area, and volume. These aforementioned measurements provide a basis for subsequent performance testing.

### 3.5. Mechanical Properties of SCPEN Films

**Figure 5:**
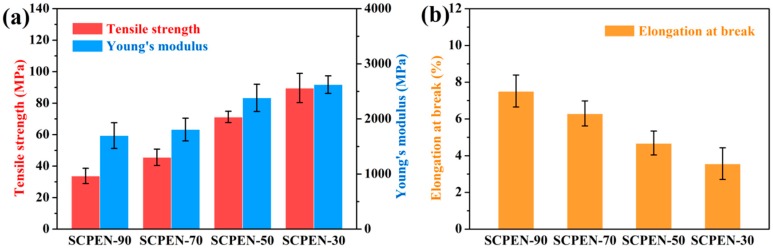
Mechanical properties of SCPEN films: (**a**) tensile strength and Young’s modulus; (**b**) elongation at break.

Adequate mechanical strength of the films is an important property of polymer-based dielectric materials. As shown in Figure 5, SCPEN-dried films show excellent mechanical properties, with the tensile strength up to 90 MPa and Young’s modulus up to 2600 MPa in the dry state. It is obvious that both tensile strength and Young’s modulus of films display a gradual upward trend as the ratio of –COOH to –SO_3_H continuously increased. Concretely, the tensile strength and Young’s modulus of SCPEN-30 films (89.6, 2622.5 MPa) are 2.6 and 1.5 times that of SCPEN-90 films (33.8, 1697.6 MPa), respectively. Meanwhile, SCPEN films display an opposite trend in elongation at break, as shown in Figure 5b. SCPEN-90 films are provided with the highest elongation at a break value of 7.52%, which means it has advantages in toughness. The content of sulfonation and carboxylation is responsible for large differences in mechanical properties for sulfonated/carboxylated copolymers, which changes the level of restriction on the mobility of polymer chains. The results above suggest that some of these copolymers are endowed with great mechanical strength to meet further applications for dielectrics.

### 3.6. Dielectric Properties of SCPEN Films

**Figure 6:**
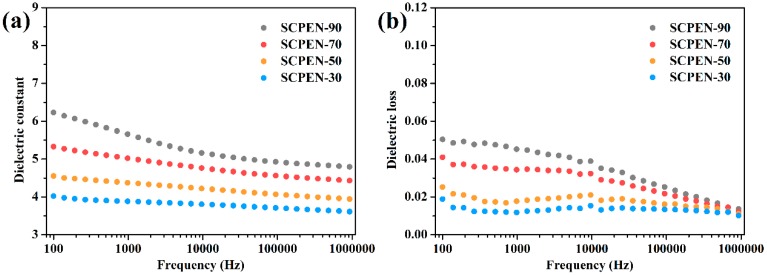
Frequency dependencies of (**a**) dielectric constant and (**b**) dielectric loss for SCPEN films.

Dielectric properties of various SCPEN films are systematically studied by multiple characterization methods. Water has a great influence on dielectric properties, so sufficient drying film is needed before testing. Figure 6 shows the dependence of the dielectric constant and loss on the frequency from 100 Hz to 1 MHz, respectively, with an applied electric field for the samples. As shown in Figure 6a, it is easy to find that the dielectric constant of SCPENs increases gradually as the ratio of –SO_3_H and –COOH increases. SCPEN-90 exhibits the highest dielectric constant (6.23 at 100 Hz). The results above are in agreement with the theory of dielectric polarization: As a result of pretreatment of long drying and relative densified structure of the film, the most likely mechanisms are the dipolar or the orientation polarization due to the nature of polar groups in the system, rather than the ionic polarization caused by water or solvents within the film [30]. At a low frequency, orientation polarization is dominant (<10^11^ Hz) because it requires the movement of permanent dipoles [31]. Compared with other parts of low dipole moments, the sulfonic groups and carboxylic groups as well as cyano groups within the SCPEN copolymer are more likely responsible for the polarization effects in the corresponding frequency range, thus generating a high dielectric constant value. Moreover, it is clear that the dielectric constants of all the films show a slight decrease with the increase of frequency, which is caused by a typical polarization relaxation process. As to SCPEN-30, the variation of its dielectric constant is only from 4.02 at 100 Hz to 3.61 at 1 MHz. Even if the mole ratio of –SO_3_H and –COOH reached 9:1, the dielectric constant is between 6.23 and 4.79. This reveals that SCPEN copolymers presented a good dielectric constant-frequency stability, which is attributed to the neutralization of several polar groups with different polarization.

The dielectric loss is the essential factor that affects the energy dissipation in practical applications. As shown in Figure 6b, the dielectric loss of the films is similar to the dielectric constant. Among the samples, although SCPEN-90 shows the highest dielectric loss (0.045 at 1 kHz), it is also at a relatively low level compared to those of the all-organic dielectric composite materials which employed conducting polymers/oligomers as fillers [32,33]. When the polarity of the system exhibits a certain degree of decline, with respect to SCPEN-50 and SCPEN-30, the dielectric loss remains basically unchanged from 100 Hz to 1 MHz (0.018 and 0.012 at 1 kHz, respectively), which is sufficient for practical applications. The increase of the dielectric loss can be attributed to the increase of dipole density on the aromatic ring of the PEN structures [34,35]. More dipoles participating in the orientation would bring about larger restriction and dielectric dissipation.

**Figure 7:**
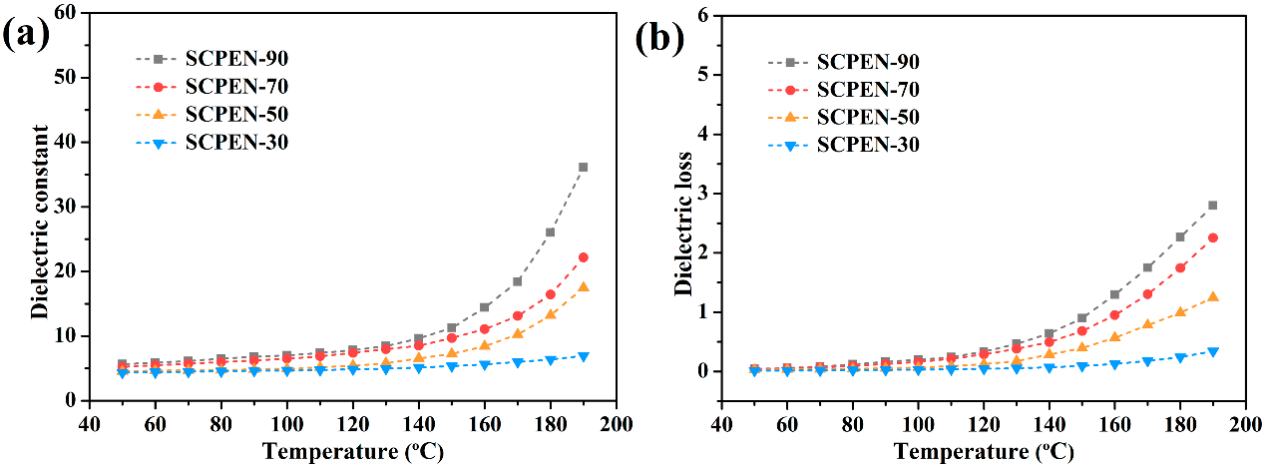
The temperature dependencies of (**a**) dielectric constant and (**b**) dielectric loss of SCPEN films at 1 kHz.

Furthermore, the dependence of the dielectric properties on the temperature is also studied at 1 kHz from 50 to 190 °C. Generally, the dielectric properties of a polymer keep relatively stable with the increase of temperature until glass transition temperature (T_g_), which is the turning point of the macromolecular motion. When T_g_ is approaching, the polymer chains promote polarization within the system as the dipole segment is gradually stretched and vigorously moved [36]. As a sort of polymer dielectric, all the SCPEN films exhibit a typical trend, as shown in Figure 7. Both the dielectric constant and dielectric loss show an abrupt increase when temperature exceeds 140 °C. It is worth pointing out that SCPEN-30 showed excellent dielectric stability at temperature below 200 °C. We speculate that this may be because SCPEN-30 has higher carboxylic acid content and therefore higher T_g_. 

**Figure 8:**
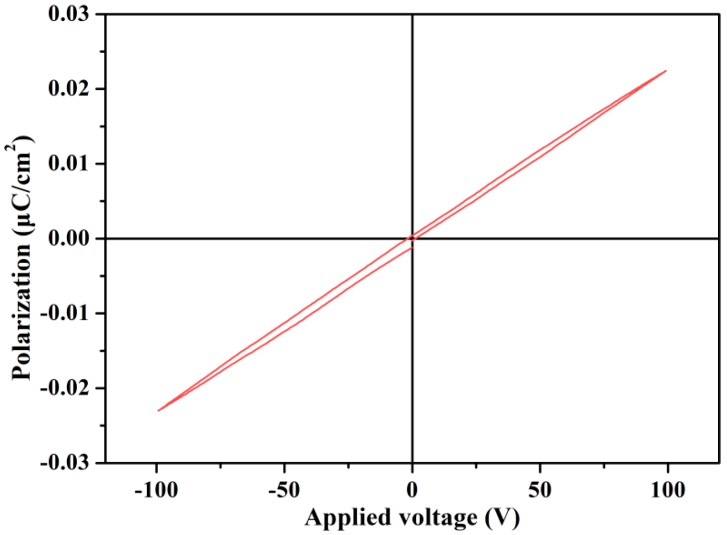
P-V loop of SCPEN film.

For polar polymers containing permanent dipoles, a typical linear dielectric polymer usually present a linear hysteresis loop [37]. Based on this, the polarization-voltage (*P*-*V*) hysteresis loop under a certain applied voltage is measured under the applied voltage of 100 V. From Figure 8, a narrow *P-V* loop confirms that SCPEN is a typical linear dielectric material. 

**Figure 9:**
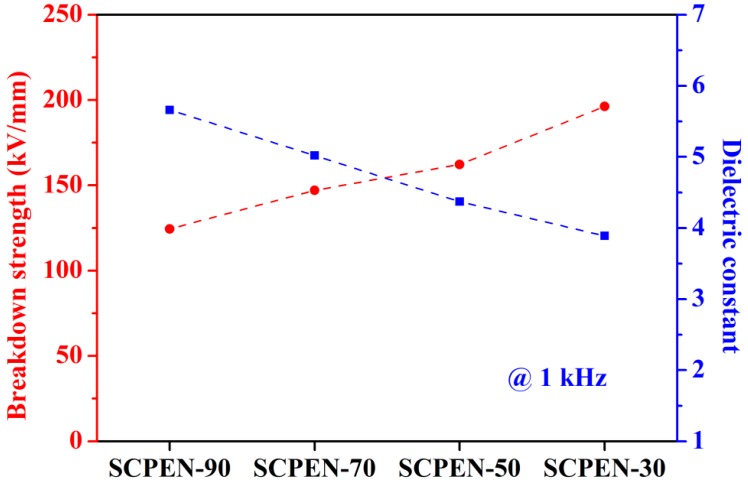
The breakdown strength and dielectric constant of SCPEN films at 1 kHz.

The electrical breakdown strength of the films is of great significance to the practical application of dielectric materials. Thus, the electrical breakdown strengths of different SCPEN films are measured in detail. The results reveal that the ratio of polar groups pending in the chain leads to a monotonous change in breakdown strength as expected. As shown in Figure 9, the breakdown strength of SCPEN films increases with the increasing –CO_2_H content, which increases from 124.4 to 196.2 kV/mm at room temperature. This phenomenon is in good agreement with the classical theory which was proposed by Stark and Garton [38]. It reveals that the breakdown strength of the polymer film is related to its tensile modulus. The breakdown strength tends to be improved by the enhancement of the tensile modulus. The high dielectric breakdown is mainly due to the absence of defects on the film, which has been confirmed by SEM.

The side groups pending on the main chain bridge the relationship between the dielectric constant and breakdown strength. On the one hand, the increasing sulfonic groups (–SO_3_H) provide opportunities for enhancing the dielectric constant for polymers, as a result of intensive orientation polarization of highly polar groups. On the other hand, carboxylic groups (–COOH) contribute to the improvement in breakdown strength. This could be attributed to the following mechanism: The presence of carboxylic groups leads to an increase in T_g_, which suppresses segmental motion and thus charge hopping or tunneling under high electric fields [39].

**Figure 10:**
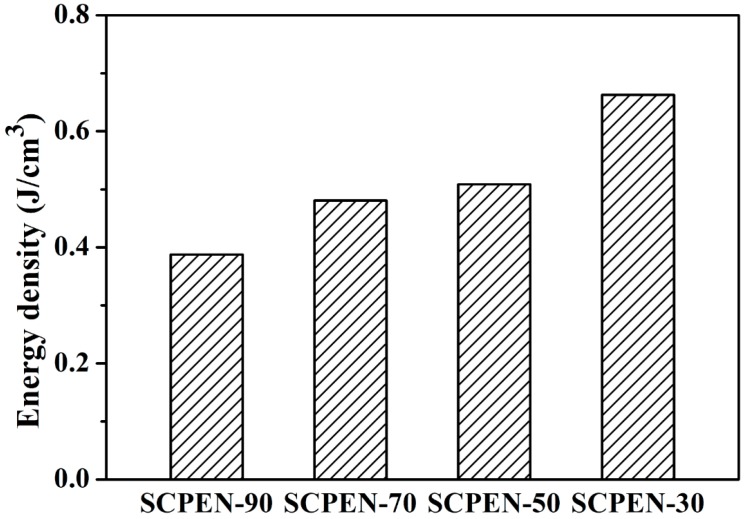
Energy density of SCPEN films at 1 kHz.

The energy density (U) of linear dielectrics is calculated by Equation (1):

(1) $$U=\frac{1}{2}\varepsilon_{0}\varepsilon_{r}E_{b}{}^{2}$$

where ε_r_ and E_b_ represent the dielectric constant and breakdown strength of the dielectric films, respectively. Based on the above results, the energy storage density of SCPEN can be obtained, as shown in Figure 10. The energy density shows a gradual increasing trend with the increase of the carboxylic group and the maximum value of 0.66 J/cm^3^ being achieved. This indicates the potential application of SCPEN films in the energy storage capacitors field.

## 4. Conclusions 

A series of poly(arylene ether nitrile) with sulfonic and carboxylic groups have been synthesized. The dielectric properties of the SCPEN copolymer was firstly evaluated in relation to its molecular structure. By adjusting the proportion of the two polar groups, the SCPENs presented an excellent dielectric constant of 4.03–6.23, dielectric loss of 0.019–0.050 at 100 Hz, and breakdown strength of 124–196 kV/mm. The increase of sulfonic groups in the SCPEN copolymer contributes to the improvement of the dielectric constant, while the introduction of carboxylic groups is beneficial for the breakdown strength, mechanical properties, and thermal stability of the films. This indicates that dielectric thin films with excellent comprehensive properties can be obtained by adjusting the appropriate proportion of sulfonic and carboxylic groups. Furthermore, this work will provide a basis for the selection of the polymer matrix, and thus promote the development of high dielectric thin films.

## Figures and Tables

**Table 1:** Components of SCPEN copolymers.

Samples	SCPEN-90	SCPEN-70	SCPEN-50	SCPEN-30
SHQ: PPL (molar)	9:1	7:3	5:5	3:7
SHQ (mol)	0.09	0.07	0.05	0.03
PPL (mol)	0.01	0.03	0.05	0.07
DFBN (mol)	0.1	0.1	0.1	0.1
K_2_CO_3_ (mol)	0.245	0.235	0.225	0.215
Yield (%)	89.2	90.4	91.5	90.8
η_inh_^a^ (dL·g^−1^)	1.15	1.11	1.10	1.15

^a^ The average value was obtained by measured at 35 °C with the concentrations of 0.5, 0.33, 0.25 g·dL^−1^ in NMP using Ubbelohde viscometer.

**Table 2:** Thermal properties of SCPENs copolymers.

Sample	SCPEN-90	SCPEN-70	SCPEN-50	SCPEN-30
*T_5%_* (°C)	317	343	369	412
*CY* (%) ^a^	55.7	59.1	61.6	63.5

^a^ Measured at 800 °C.

**Table 3:** The IEC, water uptake and swelling ratio of SCPEN films.

Films	IEC (mmol/g)	Water Uptake (%) ^a^	Swelling Ratio (%) ^a^		
		Mass	Length (S_L_)	Area (S_A_)	Volume (S_V_)
SCPEN-90	2.89	53.5	10.9	21.4	35.3
SCPEN-70	2.07	29.1	6.5	13.0	16.5
SCPEN-50	1.38	19.5	4.2	9.9	13.4
SCPEN-30	0.69	10.3	2.0	3.6	6.5

^a^ 30 °C.

## Supplementary Materials

The following are available online at https://www.mdpi.com/2073-4360/11/7/1135/s1, the details of the ion exchange capacity (IEC), water uptake (WU) and swelling ratio (SR) measurement methods for SCPEN films are displayed in the supplementary materials.
