# Supplementary material for: Component Adjustment of Poly(arylene ether nitrile) with Sulfonic and Carboxylic Groups for Dielectric Films
Source: Polymers (Basel). 2019 Jul 3;11(7):1135. doi: 10.3390/polym11071135 (PMC6681018; doi:10.3390/polym11071135)
Supplement: Supplementary file 1 [file polymers-11-01135-s001.pdf]

Supplementary Information

Component Adjustment of Poly(arylene ether nitrile) with Sulfonic and Carboxylic Groups for Dielectric Films

Chenchen Liu, Shuning Liu, Jian Lin, Lingling Wang, Yumin Huang*, Xiaobo Liu*

Research Branch of Advanced Functional Materials, School of Materials and Energy, University of Electronic Science and Technology of China, Chengdu, 611731, P. R. China

Email address: hym@uestc.edu.cn (Yumin Huang); liuxb@uestc.edu.cn (Xiaobo Liu).

Tel. /fax: +86-28-83207326.

1. Characterization

1.1 Ion exchange capacity (IEC)

The IEC of SCPEN films were determined by a typical acid-base titration. Films were dried in an oven at 100 ℃ for 6 h to obtain the weights in dry state and then soaked in 1 M NaCl solution for 24 h to substitute the H^+ ions with Na^+ ions. Then, the H^+ ions released into the solution were quantified using 0.01 M NaOH with phenolphthalein as the indicator. The IEC values were calculated using the equation below,

$$\text{IEC (mmol/g)} = \frac{V_{\text{NaOH}} \times C_{\text{NaOH}}}{W_{\text{dry}}} \quad \text{(S1)}$$

where V_{NaOH} is the consumed volume in the titration, C_{NaOH} is the concentration of the titrant, and W_{dry} is the weight of SCPEN film.

1.2 Water uptake and swelling ratio

Prior to the measurements, all of SCPEN films were dried in an oven at 100 °C for 6 h to remove the moisture, and the weights of dry films were obtained, Then, the films were soaked in deionized water at 30 °C for 24 h. The water uptake (%) was calculated by measuring the weight changes of films before and after immersion in. Equation S2 was used to calculate the water uptake of the films.

$$\text{water uptake (\%)} = \frac{W_{\text{wet}} - W_{\text{dry}}}{W_{\text{dry}}} \times 100 \quad \text{(S2)}$$

where the weights of the dry and wet films are separately represented by W_{dry} and W_{wet}.

The swelling ratio of films was obtained by measuring the changes in length, area and volume before and after immersion in water. Equations S3–S5 were used,

$$\text{Swelling in length } (S_L, \%) = \frac{L_{wet} - L_{dry}}{L_{dry}} \times 100 \qquad \text{(S3)}$$

where S_L is the swelling ratio in terms of the length (%), and the lengths of films in their wet and dry states are separately represented by L_{wet} and L_{dry}.

$$\text{Swelling in area } (S_A, \%) = \frac{A_{wet} - A_{dry}}{A_{dry}} \times 100 \qquad \text{(S4)}$$

where S_A is the swelling ratio in terms of the area (%) and the areas of films in their wet and dry states are separately represented by A_{wet} and A_{dry}.

$$\text{Swelling in volume } (S_V, \%) = \frac{V_{wet} - V_{dry}}{V_{dry}} \times 100 \qquad \text{(S5)}$$

where S_V is the swelling ratio in terms of the volume (%) and the volumes of films in their wet and dry states are separately represented by V_{wet} and V_{dry}.
